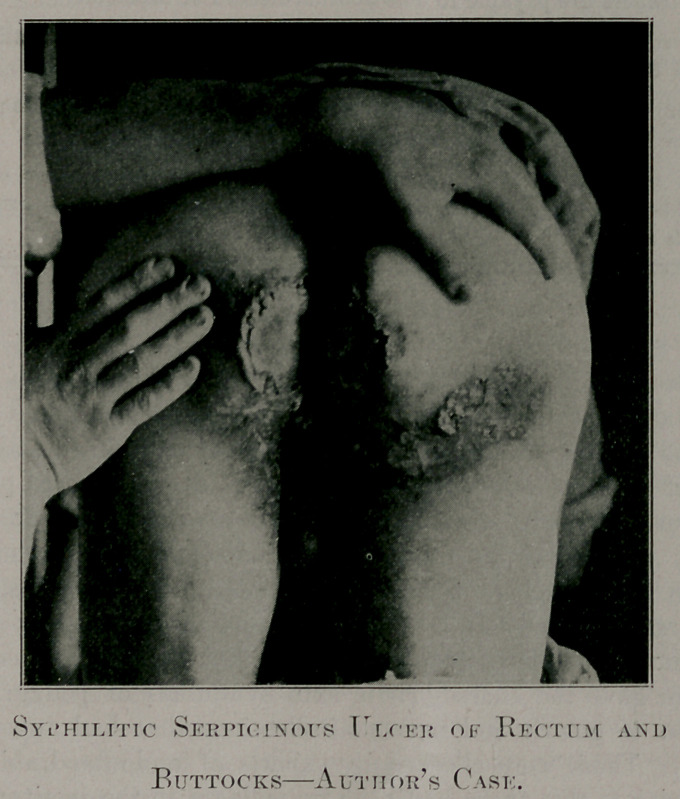# Syphilitic Serpiginous Ulceration of the Rectum and Buttocks

**Published:** 1915-12

**Authors:** E. P. Merritt

**Affiliations:** Atlanta, Ga.


### SYPHILITIC SERPIGINOUS ULCERATION OF THE RECTUM AND BUTTOCKS.

By E. F. Merritt, M. D., Atlanta, Ga.

A Greek, aged 32, came for examination 18 months ago with an ulcer in a worse condition than the illustration shows. He had mucous patches in the throat and enlargements of the inguinal, epitrochlear and post-auricular glands that usually accompany this stage of syphilis. There were loss of appetite and sleep, nervousness, loss of weight, etc. He said he had acquired a scar on the penis about a year before and the scar is still plainly to be seen.

He was given 0.5 gm. of salvarsan at once and large doses of potasium iodid and mercury were ordered. In addition he was given twice a week 1-5 grain injections of cyanide of mercury.

He improved rapidly and soon after the scar was left to show he previous ulcer. He was told, however, that he should remain under treatment for several months and until a cure could be shown by other methods. He refused further treatment.

SYPHILITIC SERPIGINOUS ULCER OF RECTUM AND BUTTOCKS—AUTHOR'S CASE.

In about four months, he returned with the symptoms very much as they were before.

This man is working in a restaurant and is a menace to all his customers because of the infectious condition of the ulcer. There should be some law whereby he could be isolated and the public protected.

924-26 Candler Bldg.

## Figures and Tables

**Figure f1:**